# Annotations

**Published:** 1909-01-02

**Authors:** 


### ANNOTATIONS.

### Academic Distinction.

Each year produces an abundant crop of highly qualified medical men, with a fine flower, as it were, of exhibitioners and gold medallists almost as unfailing. And yet the reproaches of medicine, its burden of unsolved problems and theorems, grow fewer at nothing like a corresponding rate. The various manifestations of this seeming paradox have often been pointed out. Not long ago, when medical education was being much discussed, Dr. Lauriston Shaw quoted the unceremonious dictum of an eminent surgeon who used to declare that he wanted no gold medallists as his residents. Just recently Mr. John Morgan, in a speech at a prize-giving to the students of Charing Cross Hospital, consoled the unsuccessful ones by telling them he could find no record of any one of three distinguished alumni of that school, namely, Huxley, David Livingstone, and Sir Joseph Fayrer, having gained any sort of award during their career there. It may be granted that notable instances to the contrary could be adduced, but still the fact that such a genius as Huxley's failed to impress his teachers needs explanation. Probably all the various theories to account for such cases must refer ultimately to the subconscious nature of genius, with its carelessness of practical considerations, its obedience to its own dictates and to them only, its impatience of work which is not endogenous. That this general characteristic is found also in genius for medicine, Balzac, in his wisdom knew, when he made Desplein, his famous surgeon, state in describing early struggles, that delicate minds which exercise their power in a lofty sphere are wanting in the faculty for small practical arrangements. Their talent, he says, is chance; they do not seek—they find.

### Inspection of School Children.

On December 22 the London County Council approved the recommendations of the Education Committee concerning the medical inspection of the children attending elementary schools. As a result of the consideration of 351 applications, Messrs. N. B. Harman, F.R.C.S., and P. M. Yearsley, F.R.C.S., were appointed permanent assistant medical officers in charge of ophthalmic and aural clinics respectively; and Mr. C. E. Wallis was similarly appointed for dental duties. All three of these gentlemen have been already in the employ of the council in similar capacities. It was further decided that 14 "school doctors" should be employed, 12 as quarter-time and two as half-time officials. The list of those appointed contains both consultants and general practitioners, and this is part of the systematic policy by which the appointments have been governed. The same principle has been observed in the further selection of 24 "assistant school doctors," who are to hold office for three years and to be eligible during that period for promotion to the higher rank and emoluments of the others. These officers are, like the seniors, to devote three afternoons a week to their duties. With such a staff at the disposal of the Education Committee, it is certain that the medical inspection of school children will be thoroughly and efficiently carried out in London.

